# Acoustic information masking effects of natural sounds on traffic noise based on psychological health in open urban spaces

**DOI:** 10.3389/fpubh.2023.1031501

**Published:** 2023-03-03

**Authors:** Shilun Zhang, Lin Chen

**Affiliations:** School of Architecture, Yantai University, Yantai, China

**Keywords:** traffic noise, psychological health, natural sounds, open urban space, acoustic information masking effects

## Abstract

The use of existing resources, such as natural sounds, to promote the mental health of citizens is an area of research that is receiving increasing attention. This research contributes to existing knowledge by combining a field psychological walk method and an experimental acoustic control method to compare the acoustic information masking effects of water and birdsong sounds on traffic noise based on the psychological health responses of 30 participants to such effects. The influence of traffic noise and contextual sounds on the psychological health of participants identified the potential of natural sounds in the acoustic information masking of traffic noise. Furthermore, it was found that 65.0 dBA water sounds did not mask 60.0 dBA traffic noises. However, 45.0 dBA birdsong sounds did mask it, but this effect was not significant. Additionally, contextual factors with and without crowd activity sounds were not significant in influencing psychological health through birdsong. This study contributes to public health cost savings. It may also guide the development of new ideas and methods for configuring open urban spaces according to public health needs.

## 1. Introduction

Traditionally, acoustic information masking is defined as an elevation in the perception threshold of one sound signal in the presence of other sound(s), referred to as masker(s) (1, 2). The application of acoustic information masking in a soundscape is generally described as the use of desired sounds that evoke pleasing perceptual reactions and improve soundscape quality by diverting people's attention away from target sounds (such as traffic noise) (3–5). Using the information masking effects of positive sounds on more negative ones is crucial to improve soundscape quality in open urban spaces (6). Positive sounds, such as those from water, birds, and/or wind, have different masking effects on negative sounds based on the acoustic environment experience. Among these, water sounds are frequently used as masking sounds for more annoying noises emanating from traffic or crowd activity. For example, in a case involving a fountain design in a railway station square in Sheffield, UK, a water curtain over a stainless steel noise barrier was designed to lower user discomfort from the traffic noise as well as the sounds of local crowd activity (7). In an experimental study on the annoyance of welding sound in Shanghai, researchers compared the masking effects of fountain, rain, and waterfall sounds on the noise caused by welding (5). They found that the water fountain sound was the best in terms of its masking effects on the noise caused by welding. When the sound pressure levels of the water fountain and welding sounds were equal, the annoyance of the latter was reduced by 29.0%.

Compared with water sounds, the sounds of birds have been found to be more important in improving the contextual acoustic quality of open urban spaces. For example, in an auditory experiment on emotional quality, researchers used water fountain and bird sounds to mask traffic noise (8). They found that the effects of bird sounds on traffic were better than those of water fountain sounds, as the former significantly enhanced the pleasantness and eventfulness of the soundscape. Previous studies also focused on the masking effects of positive sounds (e.g., natural sounds in open urban spaces) on traffic noise, as evaluated by the sonic environment perception (9). Various pieces of evidence have indicated that natural sounds are a potential recovery agent for psychological health (10–12), as opposed to negative ones (e.g., traffic noise), which adversely affected psychological health. However, studies assessing the masking effects of natural sounds on traffic noise based on psychological recovery are limited.

Because crowd activity sounds can exert different effects in different contexts, they are not always “bad” in all circumstances. For example, activity sounds of people at a Disney playground were found to be positive, even though residents became annoyed when they were in residential areas. Thus, crowd activity sounds can be a contextual factor for exploring soundscape quality in open urban spaces. Contextual factors in a given sonic environment are also important for soundscape quality, especially in open urban spaces. However, few studies have investigated whether sound-related contexts influence the psychological health of people (13), especially those involving activity-related sound scenarios.

In this study, acoustic information masking was defined as traffic sounds perceived incompletely and/or weakly as a result of changing natural sounds into information types (types of sound sources) and informational strength (sound pressure levels), which evoked positive emotions and cognitions. The acoustic information masking effect of traffic sounds by natural sounds was measured in terms of the psychological health values evaluated by the experimental participants (perceived psychological health). In other words, successful masking of acoustic information is defined as a situation in which the addition of natural sounds in the sonic context covered by traffic sounds improves psychological health. For example, if the participants' psychological health value increases when a 50.0 dBA birdsong sound is added to a 60.0 dBA traffic sound, based on this psychological health improvement, a 50.0 dBA birdsong will be considered to have successfully masked the acoustic information of a 60.0 dBA traffic sound.

Therefore, in this study, sound informational characteristics (including pressure levels, source types, etc.), activity-related sound contexts, social characteristics of the crowd, and psychological health were combined to answer the following research questions:

- How does traffic noise affect the mental health of people without the masking effect of contextual sounds?
- How do differences in natural sounds mask the sound information of traffic noise when considering the psychological health of people?
- What are the acoustic information masking effects of natural sounds on traffic noise in the context of the sound associated with different activities?

## 2. Materials and methods

### 2.1. Psychological field walk measurements

This field investigation aimed to establish a database regarding the effects of an acoustic environment with many sounds (contextual sounds in the field) and to examine the impact of traffic noise (in the laboratory) on mental health without the masking effect of contextual sounds. An audiovisual walk (14) with a questionnaire survey for the field acoustic measurements was used to obtain data on acoustic environmental characteristics (A-weighted sound pressure level, *L*_*Aeq*_), audiovisual experiences, and psychological response evaluations. Only audiovisual experience data were collected in the preliminary survey, while acoustic and psychological data were measured in the official investigation. Some survey points will be filtered based on the results of the audiovisual experience in the preliminary survey.

**Figure 1:**
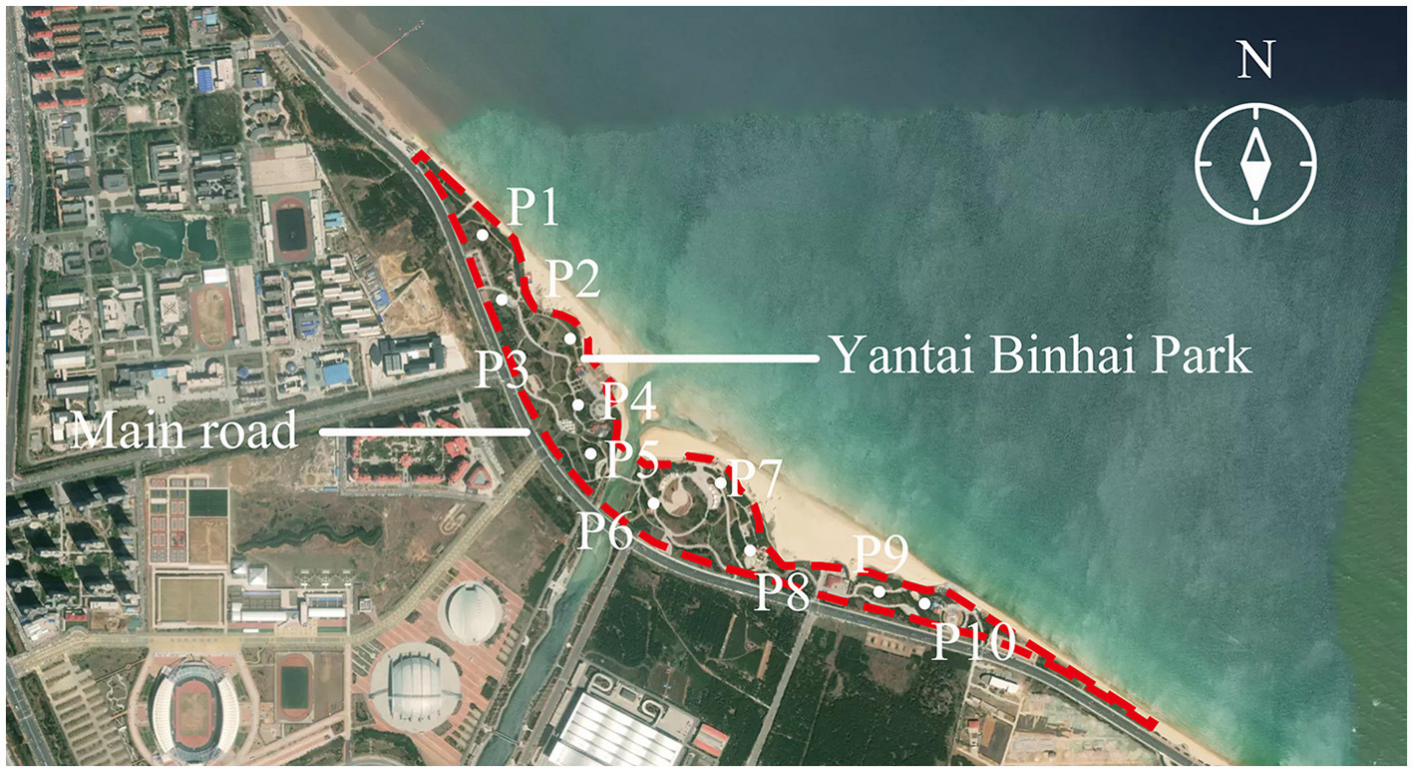
Filtered 10 survey positions in the Yantai Binhai Park (YBP).

A typical open urban space—Yantai Binhai Park (YBP) located in Yantai, China—was selected as the survey site of this study, as it includes various sound sources, such as traffic, birdsong, sea waves, and the sounds of crowd activity. Approximately 30 YBP positions were investigated by five volunteers, who all majored in Architecture and Urban Planning, for their audiovisual experiences. Data collection from these experiences aimed to identify positions in which traffic, crowd activity, and natural occurrences were dominant sources of sound in the acoustic environment to avoid the influence of any visual factors. Visual comfort (15), diversity (16), green rate (17), and space openness (15) were then used to evaluate the visual experience of these positions. Approximately 20 positions with insignificant differences in visual factors were then retained. These positions were filtered according to the perceived percentages of their domain sound sources. Volunteers were asked to evaluate the intensity of each sound source in each position according to ISO standards (18), followed by the percentage of each sound source that occupied the overall sound context. In total, 10 areas were finally defined as official measurement locations where the percentage of activity sounds, as perceived by volunteers, was < 23%. The different visual experiences of volunteers were relatively less in each of these regions than in other areas. Approximately 3 of the 10 positions were located on a main road and the other three were located on a nearby beach, while the rest were in forests. In an official survey, participants were invited to visit these 10 positions in a designated order (from P1 to P10, as shown in Figure 1). They were asked to remain in each position for 5 min and then complete the questionnaire in 2 min. Simultaneously, two volunteers were asked to measure acoustic data at each position.

A portable modular multichannel data acquisition system was used to measure *L*_*Aeq*_ in YBP, which also recorded sound samples used as audio stimuli in the laboratory. The sound pressure level meter was set to slow mode, A-weighted, 1/3 octave, and read instantaneous data every second. The sound pressure level meter probe was placed more than 1.0 m from other main reflecting surfaces and more than 1.2 m from the ground. A total of 3 min of acoustic data were obtained in each study area at YBP.

### 2.2. Experimental acoustic control measurements

**Figure 2:**
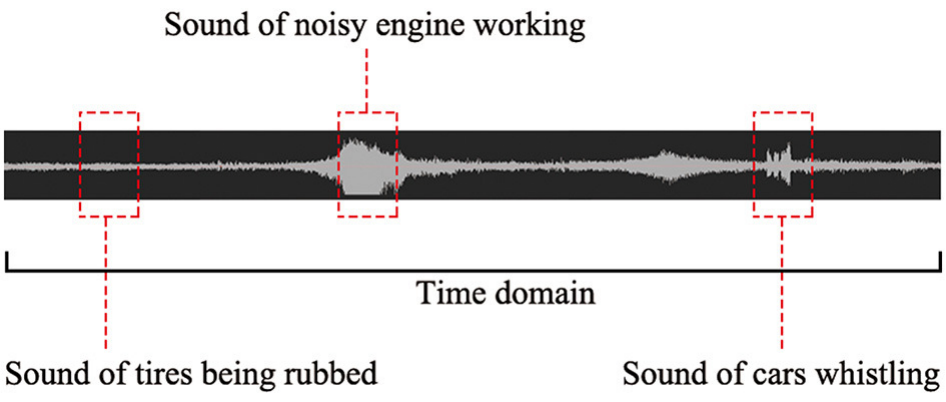
A waveform graph of traffic sounds over time domain.

Traffic, birdsong, water, and crowd activity sound samples were recorded using a recorder placed in typical urban open spaces in Yantai. The SQuadriga II recorder by the LANDTOP Company, Beijing, China, was used for this recording. A total of half an hour of traffic noise, including the sound of noisy engines, tire rubbing, and car whistling, was recorded in addition to an eight-lane two-way road adjacent to YBP. A birdsong sound was also recorded for half an hour in a natural forest with a low sound pressure level of background noise. Furthermore, because the sound of running water in a river in a park far away from the road remained consistent over time, we recorded it for only 10 min. Finally, crowd activity sounds were recorded for half an hour in a playground. To use these sound samples as input stimuli in experimental measurements, each sound was captured as a 20 s clip using software called Audition CC (2018). Regarding traffic noise, the sound of tire rubbing was selected as the input sound stimulus because the other two kinds of traffic noise were not typical in the time domain (Figure 2).

The experiment was conducted in a listening room at Yantai University. During the experiment, participants were first asked to listen to the edited sound and then complete the questionnaire reflecting psychological health. Upon completion of the experiment, they were requested to fill in their demographic characteristics.

To answer the research questions, the experiment was divided into three parts. In part one, to answer the first research question, seven levels of traffic noise (i.e., the sound of tire rubbing) were edited with different sound pressure levels (45.0, 50.0, 55.0, 60.0, 65.0, 70.0, and 75.0 dBA) using Artemis 12.0 software. The scope of *L*_*Aeq*_ was set from 45.0 to 75.0 dBA because it ranged from 45.9 to 66.5 dBA. *L*_*Aeq*_ was set to every 5.0 dBA because 10 volunteers who participated in the preliminary experiment did not feel much changes in their psychological response when *L*_*Aeq*_ was set to < 5.0 dBA. To answer the second research question, water sounds and birdsongs of different *L*_*Aeq*_ levels (45.0, 50.0, 55.0, 60.0, and 65.0 dBA) were combined with traffic noise at a level of 60.0 dBA. To answer the third research question, crowd activity sounds at a level of 60.0 dBA were added to the combination of traffic noise and natural sounds. A control test in which all 30 participants were exposed to a 60.0 dBA traffic sound and 60.0 dBA water sounds and an experimental test in which the same participants were exposed to traffic, water, and crowd activity sounds of 60.0 dBA were conducted. Water sounds and traffic noise were set at 60.0 dBA because the former should be similar to or not < 3.0 dB below the sound pressure level of the latter (19). Another experimental test was also conducted in which 30 participants were exposed to a 60.0 dBA traffic sound, 60.0 dBA birdsong sound, and 60.0 dBA crowd activity sound.

During the experiment, a speaker controlled a laptop that provided sound stimuli to the participants. However, because the software on the laptop could only control the volume of the speaker and not its *L*_*Aeq*_, the latter had to be controlled. To maintain *L*_*Aeq*_ of the sounds set by the Artemis software, as well as to ensure that *L*_*Aeq*_ participants heard the sounds consistently throughout the experiment, the speaker was debugged with a sound pressure level meter. This is because a sound pressure level meter can identify *L*_*Aeq*_ of the speaker at a specific location. For instance, when playing back the 60.0 dBA traffic audio recording with the speaker, the sound pressure level meter was set at a position where the audio would stimulate participants prior to the experiment. Then, the volume of the laptop was adjusted until the screen of the sound pressure level meter displayed 60.0 dBA. Therefore, the speaker was debugged. The distance between the participant and speaker was set at 4.0 m, with each participant participating in the experiment individually.

### 2.3. Participants and questionnaire

In total, 30 participants (aged 18–34 years; 17 men and 13 women) who majored in architecture were randomly recruited through the WeChat platform to participate in the psychological walk of this study. This age group was targeted because young people have relatively high auditory and visual sensitivity (20). They were selected according to normal vision and hearing standards because those with any impairments here would have had different experiences regarding their perceptions, thereby affecting the experimental results. Regarding the judgment of sounds within a normal hearing range, the recruits received sound stimulation recordings from a previous investigation at the survey positions in YBP. Recruits who could identify all sound types were invited to participate in the final field measurements. For the assessment of normal vision, the recruits received photos taken during the previous investigation at the survey positions in YBP. Those who could identify all colors were invited to participate in the field measurements. To reduce the influence of any psychological differences among participants, they were asked to work separately for 1 h before the experiment. In total, 30 participants who had undertaken the psychological walk were recruited again for the experiment to avoid errors and bias. The recruitment method and content were the same as in the field acoustic measurement and questionnaire survey.

This study was conducted in accordance with the Declaration of Helsinki. The method used in this study was approved by the Ethical Review Board of Yantai University (Reference: ERB2022A01). Furthermore, participants signed an informed consent form and were informed about data privacy and of their data being used only in this research.

In the questionnaire, participants were first requested to fill in their gender and age information and then to evaluate their psychological health at each position in YBP. Data on their gender and age were not required for the main research question, but they did help support the credibility of the results. Emotional and cognitive dimensions were included in the psychological health evaluation. Four pairs of adjectives with opposite meanings were used to evaluate the psychological health of participants (14), including cheerful–depressed (CD), relaxed–anxious (RA), energetic–fatigued (EF), and focused–distracted (FD). Among these, CD and RA were used to evaluate the emotional dimensions of psychological health, while EF and FD were used to evaluate the cognitive dimensions. A nine-point bipolar scale from −4 (negative psychology) to +4 (positive psychology) was used to quantify these four indicators (Table 1). Finally, the averages of CD and RA were used to represent the psychological health of participants at the emotional level and, correspondingly, the averages of EF and FD were used to represent the psychological health of participants at the cognitive level. Then, the language in the psychological questionnaire was translated from English to Chinese by a Chinese graduate who majored in English and back-translated by a fluent speaker of both languages. A great similarity was established between the English and Chinese versions. Before the official experiment, the questionnaire was pretested with undergraduates to ensure that children could easily and accurately comprehend the items.

**Table 1:** Questionnaire survey content on psychological health.

**Assessment of your mental health state**										
Depressed	−4	−3	−2	−1	0	1	2	3	4	Cheerful
Anxious	−4	−3	−2	−1	0	1	2	3	4	Relaxed
Fatigued	−4	−3	−2	−1	0	1	2	3	4	Energetic
Distracted	−4	−3	−2	−1	0	1	2	3	4	Focused

### 2.4. Statistical analysis

SPSS 20.0 was used to analyze the data obtained from the field and experimental acoustic measurements and the questionnaires. First, the normality of the sample was analyzed with a one-sample Kolmogorov–Smirnov test. The results show that the total sample follows a normal distribution because all *p*-values were >0.05. Therefore, the sample size of this study was statistically significant. Second, Pearson's correlation was used to analyze the relationship between *L*_*Aeq*_ and psychological health values in both field and experimental studies, respectively, because of the sample normality, the continuity of the variables, and the linearity of the variables. Additionally, it was used to calculate the relationship between age and psychological health value. An independent sample *t*-test was used to analyze the influence of gender on the psychological health of participants. Further, a *t*-test set at a *p*-value of < 0.05 was used to test for any significant differences. Third, a linear regression analysis was used to calculate the relationships between *L*_*Aeq*_ and psychological health values in both field investigations and experimental measurements. The *t*-test was used to test the significance of the regression coefficient, while an *F*-test was used to verify the significance of the regression equation. Fourth, a paired-sample *t-*test analysis was performed to compare the differences between the information masking effects of various natural sounds on traffic noise based on psychological health values. Moreover, the confidence interval (CI) was set to 95%. The acoustic information-making effect of natural sounds was considered to be significant as the results of the paired-sample *t-*test between the control and experimental tests showed that *p*-values were < 0.05. Finally, the reliability and validity of the questionnaire were measured using reliability testing and factor analyses, respectively. The Cronbach's alpha and Kaiser–Meyer–Olkin (KMO) values in the SPSS software were used to evaluate the reliability and validity of the psychological questionnaire, respectively. The α-value of the questionnaire in the field and experimental studies was 0.884 and 0.892, respectively, which means that the questionnaire was appropriate. The KMO values of the questionnaire in the field and experimental measurement were 0.755 and 0.775, respectively, which means that the questionnaire was valid.

## 3. Results

Regarding the influence of gender and age on the psychological health of participants, according to the results from the field psychological walk measurement, there was no significant difference between men and women in the emotional dimension (*p* = 0.952), while there was a significant difference in the cognitive dimension (*p* = 0.020). It was found that age was negatively correlated with the psychological health value (*p* = 0.016 in the emotional dimension and *p* = 0.000 in the cognitive dimension). The results of the experimental acoustic control measurement indicated that there were no significant differences between men and women in both emotional (*p* = 0.000) and cognitive (*p* = 0.000) dimensions. It was determined that age was not correlated with the psychological health value (*p* = 0.152 in the emotional dimension and *p* = 0.192 in the cognitive dimension).

### 3.1. Different effects of traffic noise and contextual sounds on psychological health

**Figure 3:**
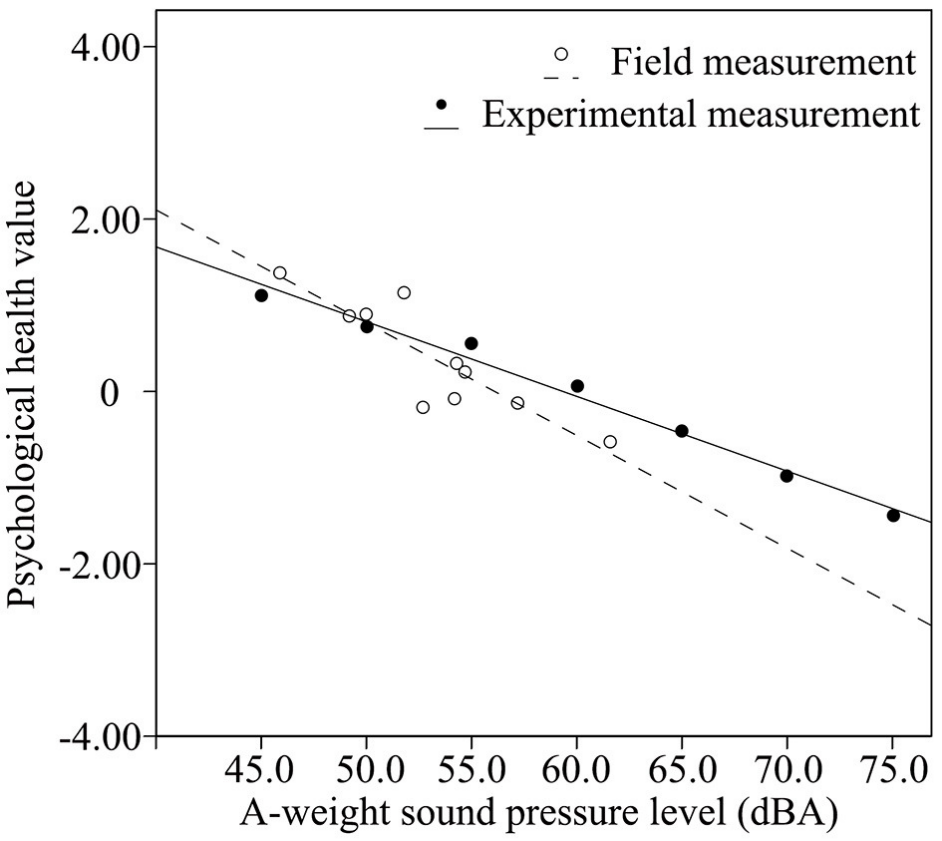
Relationship between *L*_*Aeq*_ and psychological health values within the emotional dimension.

**Figure 4:**
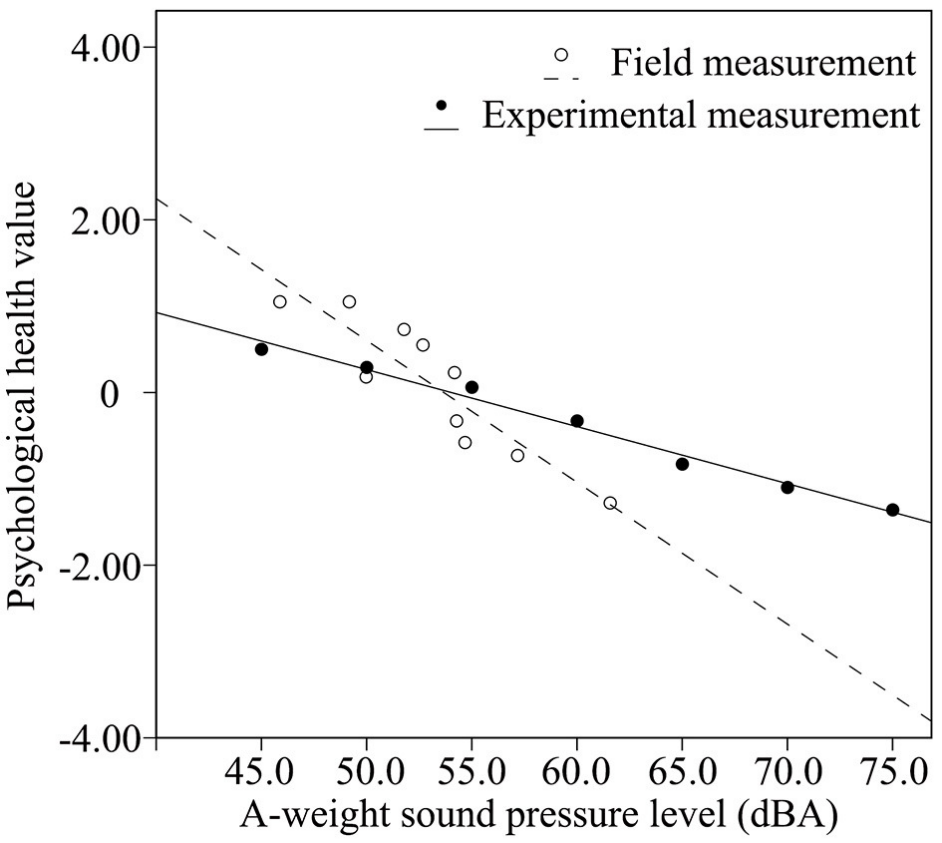
Relationship between *L*_*Aeq*_ and psychological health values within the cognitive dimension.

According to the results of our Pearson's correlation analysis, the relationships between *L*_*Aeq*_ and the psychological health values, in both the field and experimental studies, were negatively correlated (*p* < 0.01). Figures 3, 4 outline the trends in the relationship between *L*_*Aeq*_ and psychological health values in both fields (emotional dimensions in Figure 3: *R*^2^ = 0.690, *p* = 0.001 and cognitive dimensions in Figure 4: *R*^2^ = 0.823, *p* = 0.000) and experimental studies (emotional dimensions: *R*^2^ = 0.986, *p* = 0.000 and cognitive dimensions: *R*^2^ = 0.986, *p* = 0.000). In the field study, *L*_*Aeq*_ represents the total A-weighted level of all contextual sounds, including traffic noise, natural sounds, and non-dominant crowd activity sounds. However, *L*_*Aeq*_ in the experimental study refers to the A-weighted sound pressure level of traffic noise without any other contextual sounds. When *L*_*Aeq*_ was increased, we found a decrease in psychological health values due to its influence on both contextual sounds and traffic noise. When *L*_*Aeq*_ of traffic noise was increased by 5.0 dBA, psychological health values were decreased by 0.435 in the emotional dimension and 0.330 in the cognitive dimension.

A significant difference was found between traffic noise and contextual sounds in their effect on the measured psychological health values. Within the emotional dimension (Figure 3), when the A-weighted sound pressure level was < 50.1 dBA, the psychological health level in the field measurement was higher than that of the experimental measurement with the same *L*_*Aeq*_. When *L*_*Aeq*_ was more than 50.1 dBA, the psychological health value declined faster in the field measurement than in the experimental measurement. Furthermore, the psychological health value along the emotional dimension tended to be negative in the field measurement when *L*_*Aeq*_ reached 56.1 dBA, while it was also negative in the experimental measurement when *L*_*Aeq*_ reached 59.1 dBA, because participants could not bear the acoustic context involving three sounds with a higher *L*_*Aeq*_.

In terms of the cognitive dimension (in Figure 4), when the A-weighted sound pressure level was lower than 53.6 dBA, the psychological health level was also higher in the field measurement than in the experimental measurement with the same *L*_*Aeq*_. Further, when *L*_*Aeq*_ was more than 53.6 dBA, the psychological health value declined faster in the field measurement than in the experimental measurement. Finally, psychological health values within the cognitive dimension tended to be negative in the field measurement and in the experimental measurement when *L*_*Aeq*_ reached 53.8 dBA and reached 54.1 dBA, respectively.

### 3.2. Information masking effect of natural sounds

In the experimental measurement, one control test and five experimental tests were established. The control test involved the use of a 60.0 dBA traffic noise as the experimental stimulus, while the five experimental tests involved the addition of 45.0, 50.0, 55.0, 60.0, and 65.0 dBA water sounds to the control test conditions as experimental stimuli. In this study, a 60.0 dBA traffic noise was used because it was close to the total sound pressure level in a field traffic environment. All 30 participants received sound stimulation from each test, including one control test and five experimental tests, and then completed the questionnaire regarding their psychological health states (Tables 2, 3). Our results show that water sounds at 45.0–65.0 dBA do not mask the information of traffic noise because the average psychological health level of experimental test 1 was less than that of the control test.

**Table 2:** Comparisons between the psychological health values on the emotional dimension in the control test and experimental test 1.

**Control test**	**Experimental test 1**	**Mean difference**	**Standard error**	***p***	**95% confidence interval**	
					**Lower limit**	**Upper limit**
60.0 dBA traffic noise	45.0 dBA water sound + 60.0 dBA traffic noise	0.219	1.016	0.232	−0.147	0.585
	50.0 dBA water sound + 60.0 dBA traffic noise	0.109	0.877	0.486	−0.207	0.426
	55.0 dBA water sound + 60.0 dBA traffic noise	0.391^*^	0.957	0.028	−0.045	0.736
	60.0 dBA water sound + 60.0 dBA traffic noise	0.531^*^	1.270	0.024	0.074	0.989
	65.0 dBA water sound + 60.0 dBA traffic noise	0.766^*^	1.356	0.003	0.277	1.255

^*^p-value < 0.05.

**Table 3:** Comparisons between the psychological health values on the cognitive dimension in the control test and experimental test 1.

**Control test**	**Experimental test 1**	**Mean difference**	**Standard error**	***p***	**95% confidence interval**	
					**Lower limit**	**Upper limit**
60.0 dBA traffic noise	45.0 dBA water sound + 60.0 dBA traffic noise	0.375^*^	1.008	0.044	−0.173	0.923
	50.0 dBA water sound + 60.0 dBA traffic noise	0.359	1.010	0.053	−0.189	0.907
	55.0 dBA water sound + 60.0 dBA traffic noise	0.328	0.964	0.063	−0.220	0.876
	60.0 dBA water sound + 60.0 dBA traffic noise	0.406	0.201	0.052	−0.142	0.954
	65.0 dBA water sound + 60.0 dBA traffic noise	0.578^*^	0.255	0.031	0.030	1.126

^*^p-value < 0.05.

The results of a paired-sample *t-*test in Table 2 show that the mean difference between the control test and experimental test 1 was significant when *L*_*Aeq*_ of water sounds on traffic noise was >55.0 dBA in terms of the emotional dimension. This indicates that the information masking effect of water sounds was significantly lower when its *L*_*Aeq*_ was >60.0 dBA. In terms of the cognitive dimension, Table 3 presents a significant mean difference between the control test and experimental test 1 when *L*_*Aeq*_ of water sounds was 45.0 and 65.0 dBA, which indicates that the information masking effect of water sounds on traffic noise was not significantly less when *L*_*Aeq*_ of the former ranged between 50.0 and 60.0 dBA.

Regarding the information masking effects of birdsong on traffic noise, the mean difference results of a paired-sample *t-*test in Tables 4, 5 indicated that the average value of participants' psychological health increased, including both the emotional and cognitive dimensions, when birdsongs with different *L*_*Aeq*_ were added in the acoustic environment involving a 60.0 dBA traffic noise. However, there were no significant differences in information masking effects among birdsong sounds of different *L*_*Aeq*_ on traffic noise because their *p*-value was >0.05. Therefore, based on psychological health responses, birdsong sounds do mask the traffic noise information in spite of its non-significance.

**Table 4:** Comparisons between the psychological health values on the emotional dimension in the control test and experimental test 2.

**Control test**	**Experimental test 2**	**Mean difference**	**Standard error**	***p***	**95% confidence interval**	
					**Lower limit**	**Upper limit**
60.0 dBA traffic noise	45.0d BA birdsong + 60.0 dBA traffic noise	−0.234	1.000	0.195	−0.595	0.126
	50.0 dBA birdsong + 60.0 dBA traffic noise	−0.172	1.075	0.373	−0.559	0.216
	55.0 dBA birdsong + 60.0 dBA traffic noise	−0.250	1.257	0.269	−0.703	0.203
	60.0 dBA birdsong + 60.0 dBA traffic noise	−0.188	1.674	0.531	−0.791	0.416
	65.0 dBA birdsong + 60.0 dBA traffic noise	−0.422	1.792	0.193	−1.068	0.224

^*^p-value < 0.05.

**Table 5:** Comparisons between the psychological health values on the cognitive dimension in the control test and experimental test 2.

**Control test**	**Experimental test 2**	**Mean difference**	**Standard error**	***p***	**95% confidence interval**	
					**Lower limit**	**Upper limit**
60.0 dBA traffic noise	45.0 dBA birdsong + 60.0 dBA traffic noise	−0.094	1.058	0.620	−0.475	0.288
	50.0 dBA birdsong + 60.0 dBA traffic noise	−0.063	1.162	0.763	−0.482	0.357
	55.0 dBA birdsong + 60.0 dBA traffic noise	−0.109	1.162	0.598	−0.528	0.310
	60.0 dBA birdsong + 60.0 dBA traffic noise	−0.250	1.503	0.354	−0.791	0.292
	65.0 dBA birdsong + 60.0 dBA traffic noise	−0.172	1.625	0.554	−0.758	0.414

^*^p-value < 0.05.

### 3.3. Influence of crowd activity sounds in the acoustic context

The results showed that the average psychological health value decreased by 0.160 in the emotional dimension and 0.560 in the cognitive dimension when a 60.0 dBA crowd activity sound was added in the sonic environment with a 60.0 dBA traffic noise and a 60.0 dBA water sound. A paired-sample *t-*test was used to compare the mean differences of the control test with crowd activity sound and the experimental test without crowd activity sound, as samples from both tests followed a normal distribution, and the *t*-test results were from the same participants. The results from a paired-sample *t-*test indicated a significant difference in psychological health before and after adding a 60.0 dBA activity sound (*p* = 0.005 in the emotional dimension and *p* = 0.000 in the cognitive dimension). Therefore, the information masking effect of water sounds decreased after adding a 60.0 dBA activity sound in an acoustic context characterized by 60.0 dBA water sounds and a 60.0 dBA traffic noise. Additionally, there were significant differences between the information masking effect of water sounds on traffic noise in a sonic environment with and without crowd activity sound.

The information masking effect of birdsong on traffic noise was compared in an acoustic context with and without crowd activity sound. The results showed that the mean value of psychological health decreased by 0.375 in the emotional dimension and 0.735 in the cognitive dimension after adding a 60.0 dBA crowd activity sound. A paired-sample *t*-test was used to compare mean differences between the control test with the crowd activity sound and the experimental test without the crowd activity sound because the samples from both tests followed a normal distribution, and the *t*-test results were from the same participants. The results from a paired-sample *t-*test indicated no significant differences in psychological health before and after adding a 60.0 dBA crowd activity sound (*p* = 0.340 in the emotional dimension and *p* = 0.750 in the cognitive dimension). Therefore, the information masking effect of birdsong decreased after adding a 60.0 dBA activity sound in an acoustic context characterized by a 60.0 dBA birdsong sound and 60.0 dBA traffic noise. Additionally, there were no significant differences between the information masking effect of birdsong sounds on traffic noise in a sonic environment with and without crowd activity sound.

## 4. Discussion

Regarding the different effects of traffic noise and contextual sounds on psychological health, we determined that the A-weighted sound pressure level was < 50.1 dBA; the psychological health value in the field measurement was higher than that in the experimental measurement with the same *L*_*Aeq*_. It was found that natural sounds in the field measurement could mask traffic sounds because they could divert participants' attention away from traffic sounds, thereby increasing the psychological health value of participants. To identify whether natural sounds could mask the acoustic information of traffic sounds, we designed a series of acoustic control experiments. By adding water sounds in the acoustic context with only traffic sounds, the level of psychological health of participants was reduced. By adding birdsong sounds in an acoustic context with only traffic sounds, the level of psychological health of participants increased, but the increment was not evident.

Therefore, the abovementioned results revealed that water sounds could not mask the information of traffic noise, but the birdsong could mask in spite of its non-significance. The addition of water sounds can still improve soundscape quality, as reflected in the comfort experienced in an acoustic context of traffic and crowd activity sounds as the dominant sound sources, which has been documented in a previous study (7). For example, in the acoustic context of a central station square in Sheffield, UK, crowd activity and traffic were the dominant sound sources, but adding fountain water sounds increased the soundscape assessment. However, the results of this study demonstrate that water sounds cannot improve soundscape quality, as indicated by the psychological health values of our respondents, which is consistent with the results proving that the use of water sounds to mask traffic noise is unreliable (21).

We analyzed the reasons why the results of this study were different from previous results, which could be understood as different contextual factors resulting in different effects of water sounds in the acoustic context. To examine whether the effects of water sounds varied significantly by contextual factors, we compared acoustic information masking effects of water sounds in the acoustic context with and without a crowd activity sound. The results indicate a significant difference between before and after adding a crowd activity sound. Therefore, the effects of water sounds in this study are different from those found in previous studies, probably due to contextual factors not limited to the activity sound.

This study has some limitations. For example, water sounds used in this experiment were recorded from a flowing river; however, sound pressure levels distributed in the frequency bands of other water sounds, such as fountains, waterfalls, and tides, may be different from those of a flowing river, resulting in different effects. Therefore, other types of water sources, like fountain, waterfall, and tidal sounds, should also be considered in future research. Furthermore, music (22, 23) might also have a positive influence on people's psychological health. Thus, the information masking effects of music on traffic noise should also be investigated in future research. In addition, long-term acoustic stimulation (24) seems to be helpful for determining the potential of water sounds on improving users' perception in an open-plan office. This should be considered in further experiments.

## 5. Conclusion

This study integrated field and experimental methods to compare the acoustic information masking effects of water and birdsong sounds on traffic noise based on psychological health responses. This study found that psychological health values are dependent on the context of traffic acoustics and the sound environment. Herein, we found that water sounds could not mask traffic noise. However, it could be masked by birdsong sounds in spite of its non-significance. Furthermore, the acoustic information masking effects of birdsong did not change significantly in the acoustic context with or without crowd activity sounds.

## Data availability statement

The original contributions presented in the study are included in the article/supplementary material, further inquiries can be directed to the corresponding author.

## Ethics statement

The studies involving human participants were reviewed and approved by Yantai University Ethical Review Board. The patients/participants provided their written informed consent to participate in this study.

## Author contributions

SZ: term, conceptualization, methodology, software, validation, formal analysis, investigation, resources, data curation, writing—original draft, writing—review and editing, visualization, supervision, and project administration. LC: writing—review and editing. Both authors contributed to the article and approved the submitted version.
